# Supply- and Demand-Side Factors Influencing Utilization of Infant and Young Child Feeding Counselling Services in Viet Nam

**DOI:** 10.1371/journal.pone.0151358

**Published:** 2016-03-10

**Authors:** Phuong H. Nguyen, Sunny S. Kim, Tuan T. Nguyen, Lan M. Tran, Nemat Hajeebhoy, Edward A. Frongillo, Marie T. Ruel, Rahul Rawat, Purnima Menon

**Affiliations:** 1 Poverty, Health, and Nutrition Division, International Food Policy Research Institute, Washington, DC, United States of America; 2 FHI 360, Hanoi, Viet Nam; 3 Department of Health Promotion, Education, and Behavior, University of South Carolina, Columbia, SC, United States of America; University of São Paulo, BRAZIL

## Abstract

Adequate utilization of services is critical to maximize the impact of counselling on infant and young child feeding (IYCF), but little is known about factors affecting utilization. Our study examined supply- and demand-side factors associated with the utilization of IYCF counselling services in Viet Nam. We used survey data from mothers with children <2y (n = 1,008) and health staff (n = 60) from the evaluation of a program that embedded IYCF counseling into the existing government health system. The frequency of never users, one-time users, repeat users, and achievers of the recommended minimum number of visits at health facilities were 45.1%, 13.0%, 28.4% and 13.5%, respectively. Poisson regression showed that demand-generation strategies, especially invitation cards, were the key factors determining one-time use (Prevalence ratio, PR 3.0, 95% CI: 2.2–4.2), repeated use (PR 3.2, 95% CI: 2.4–4.2), and achievement of minimum visits (PR 5.5, 95% CI: 3.6–8.4). Higher maternal education was associated with higher utilization both for one-time and repeated use. Being a farmer, belonging to an ethnic minority, and having a wasted child were associated with greater likelihood of achieving the minimum recommended number of visits, whereas child stunting or illness were not. Distance to health center was a barrier to repeated visits. Among supply-side factors, good counselling skills (PR: 1.3–1.8) was the most important factor associated with any service use, whereas longer employment duration and greater work pressure of health center staff were associated with lower utilization. Population attributable risk estimations showed that an additional 25% of the population would have achieved the minimum number of visits if exposed to three demand-generation strategies, and further increased to 49% if the health staff had good counseling skills and low work pressure. Our study provides evidence that demand-generation strategies are essential to increase utilization of facility-based IYCF counselling services in Viet Nam, and may be relevant for increasing and sustaining use of nutrition services in similar contexts.

## Introduction

Best practices in infant and young child (IYCF) feeding are essential for improving the nutritional status of children and promoting healthy growth and development [1–3]. Yet both breastfeeding and complementary feeding practices remain suboptimal in developing countries, where the prevalence of exclusive breastfeeding is about 39% [4]. In Viet Nam, <20% of infants are exclusively breastfed in the first 6 months, and complementary foods are often introduced too early and tend to be of poor quality [5].

High-quality and timely IYCF counselling has been shown to be an effective strategy to improve both child feeding practices and the nutritional status of children [6–8]. Community-based initiatives using peer counsellors have been successful in supporting exclusive breastfeeding in several countries [9, 10]. Also, the delivery of clear educational messages on complementary feeding practices which emphasize the importance of hygienic, high-energy and appropriate home-prepared foods, coupled with the timely introduction of complementary foods, has been shown to lead to significant gains in the height and weight of children in low and middle-income countries [11].

Scaling up nutrition interventions to achieve adequate delivery, coverage, and utilization often requires integration with established delivery platforms, such as government health systems. However, health systems worldwide face challenges in improving service access and utilization [12], influenced by resource availability, service quality, cost, health beliefs and other user characteristics. As health systems increase their focus on nutrition to prevent poor health outcomes [13], further challenges may be seen in the delivery of preventive services that are not perceived as an immediate threat to illness [14] and require repeated use for sustained behavior change. Therefore, studies of the drivers of service utilization are needed to understand how to improve access and utilization of specific health services, particularly preventive services such as nutrition education.

Until recently in Viet Nam, government nutrition programs had not systematically integrated IYCF counselling services into routine health services [15, 16]. Health facilities lacked personnel to provide counselling services and adequate materials and infrastructure (e.g., a separate counselling room and sufficient guidelines and educational materials to support breastfeeding practices) [17]. In addition, health providers often had outdated information about optimal IYCF practices and lacked sufficient training and practical guidance on how to provide counselling to mothers. The quality, frequency, and follow-up of in-service training on IYCF was also poor [17].

To address these gaps, Alive & Thrive (A&T), an initiative aimed at improving suboptimal IYCF practices, applied the principles of social franchising to enhance IYCF counselling services in Viet Nam [18]. Incorporating elements of social franchising into government health facilities significantly improved the quality of nutrition counselling services, particularly the physical infrastructure (availability of counselling room and materials) and service delivery (increased knowledge by health staff of optimal breastfeeding practices, and improved technical content and interpersonal communication skills during counselling), resulting in high client satisfaction [19]. The services were incorporated into the functional and well-used government health system in Viet Nam [20], which provided a stable service-delivery environment. Utilization of the facility-based counseling services was lower than expected, however, one year after the launch of the first franchise [19, 21] as well as at endline (2 years later). This paper therefore examines the supply- and demand-side factors that influenced the utilization of IYCF counselling services in Viet Nam and highlights the key factors that, if strengthened, could markedly improve service utilization in this context.

### Program description

Since 2009, FHI 360 and Save the Children in partnership with NIN and provincial authorities developed and implemented a social franchise model branded as Mat Troi Be Tho (MTBT), or Little Sun in English, across 15 provinces in Viet Nam. MTBT has two main purposes: 1) to standardize and monitor services to ensure that IYCF counselling is uniform and of good quality; and 2) to build on the existing healthcare infrastructure and decentralized services to ensure utilization and sustainability. Details of the MTBT have been described elsewhere [21]; a short summary is provided below.

The MTBT franchise operates through health facilities at various levels, but the most comprehensive package of services is delivered in commune health centers (CHCs) and includes five major services: 1) promotion of exclusive breastfeeding (EBF) during the third trimester of pregnancy, 2) support of EBF at the time of delivery, 3) management of EBF during the first 6 months of life, 4) education on complementary feeding (CF) during 4 to 5.9 months of age, and 5) CF management during 6 to 24 months of age. Providers deliver the services through individual and group counselling. Based on this comprehensive package, each mother-child pair is expected to receive at least 9 counselling contacts over a 27-month period (with a maximum of 15 contacts by intervention design), i.e., at least 4 contacts by 6 months, 7 contacts by 12 months, and 9 contacts by 24 months of child age. This package of recommended minimum contacts is based on the milestones for child feeding and nutrition deemed important for a child to achieve optimal growth and development in the first 1,000 days of life.

Demand for franchise services is generated through ongoing delivery of invitation cards to pregnant women or mothers with children <24 months of age by village health workers or local nutrition collaborators, dissemination and display of promotional print materials, organization of promotional events, and use of mass media channels. To promote optimal IYCF practices and the MTBT franchise use, A&T initiated a mass-media campaign six months after the first franchise model was launched. To date, the MTBT franchise has been introduced in nearly 1,000 government health centers, a majority within CHCs.

## Methods

### Data sources and study population

**Fig 1:**
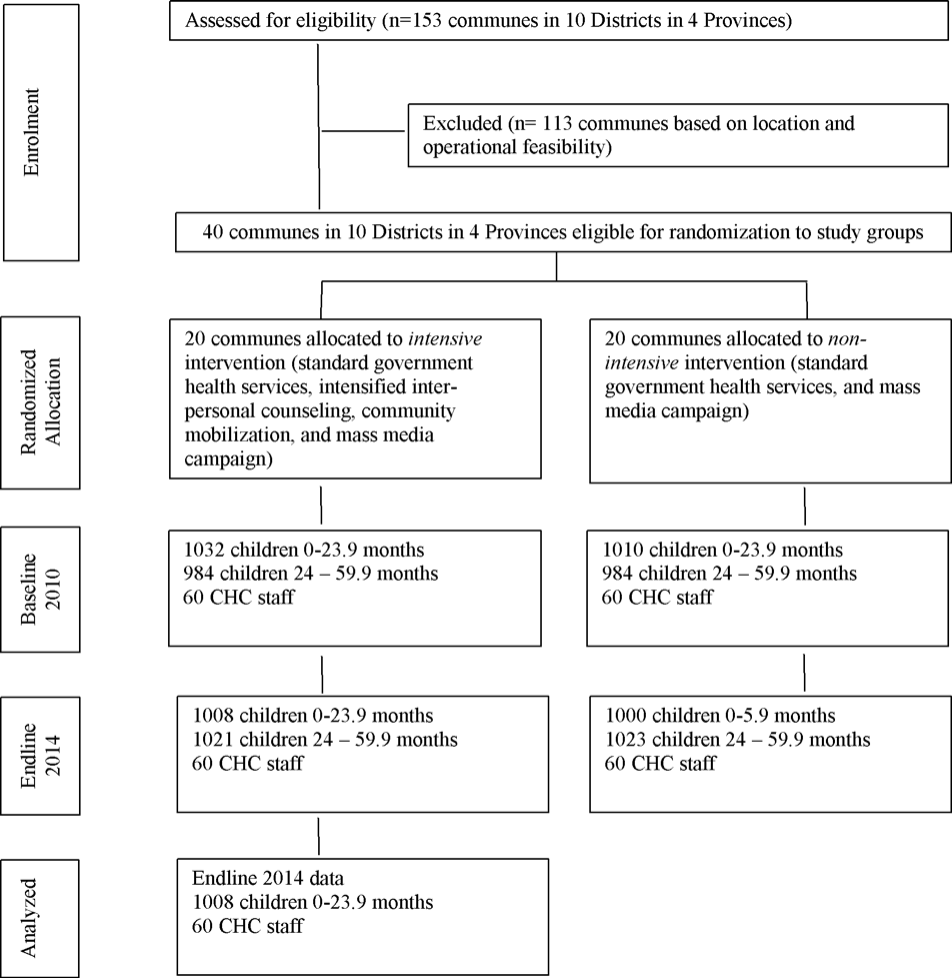
Trial Profile.

Data used for this study were from household and health staff surveys which were conducted in the context of a cluster-randomized impact evaluation to compare the impact of two Alive & Thrive (A&T) intervention packages in Vietnam, i.e., an intensive package consisting of intensified interpersonal counseling and mass media compared to a non-intensive package consisting of standard nutrition counseling along with mass media [22]. The endline survey covered 4,052 households and 120 commune health staff in 40 communes in four provinces (Thai Nguyen, Thanh Hoa, Quang Ngai and Vinh Long) (Fig 1), which span the northern, central, and southern regions of Viet Nam and are geographically representative of the 15 provinces where A&T operates. All households with children under 5 years and their mothers in study communes were invited to participate in the survey. There were no exclusion criteria. Refusals to participate were not counted but were minimal. For the staff survey, 3 out of the 5–6 staff members in each commune were selected for interview. These included the commune doctor, the midwife, and the nurse in charge of nutrition activities.

Data collection was carried out between June and August 2014, about 36 months after the launch of the first MTBT franchise. In addition to the household and health worker surveys (both using face-to-face interviews with structured questionnaires), structured observations of counseling sessions were also conducted.

This paper focuses on the factors influencing service utilization within MTBT franchises; therefore, analyses were restricted to 1,008 mother-child dyads (with a child up to 24 months of age) and 60 CHC staff in the MTBT areas only. Ethical approval was obtained from the Institutional Review Board of the Institute of Social and Medical Studies in Hanoi, Viet Nam and International Food Policy Research Institute, USA. Written informed consent was obtained from all mothers.

### Conceptual framework for service utilization

**Fig 2:**
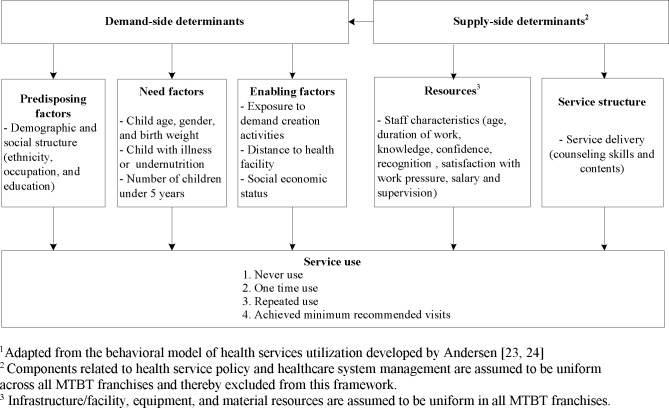
Conceptual framework of determinants of IYCF counseling services utilization. ^1^Adapted from the behavioral model of health services utilization developed by Andersen [23, 24]^. 2^ Components related to health service policy and healthcare system management are assumed to be uniform across all MTBT franchises and thereby excluded from this framework. ^3^ Infrastructure/facility, equipment, and material resources are assumed to be uniform in all MTBT franchises.

The selection of factors potentially associated with service utilization was guided by the conceptual framework (Fig 2) adapted from the behavioral model of health services utilization developed by Andersen [23, 24]. This model has been used widely for empirical research on utilization of maternal health services [25–27], integrated community case management of childhood illness [28], and medical care [29]. The model proposed that use of health services is a function of characteristics of both individual user and the health system. In our study, we considered the user characteristics as demand-side factors with three components: predisposing characteristics, enabling factors, and need factors. Characteristics of the health care system are supply-side factors, which include the health service policy, service structure, and resources.

### Dependent variables

The frequency of service use was counted by total visits from birth. We identified four categories of service use to characterize the primary outcome of service utilization: never used, one-time use, repeated use, and achieved minimum number of visits (according to child age and program recommendation). One-time users are likely drawn to try out the service mainly due to demand-side factors such as personal curiosity, awareness of the service, perceived need or susceptibility [24], and personal characteristics such as age, education. and socioeconomic status [14]. Repeat users and achievers of the minimum number of visits may be driven by both demand-side factors (e.g., service reminders and perceived need based on child’s nutrition and health status) and supply-side factors (e.g. health staff characteristics and service quality) [24, 30].

### Independent variables

#### Demand-side factors

Predisposing characteristics represent the tendency that an individual is more or less likely to use health services based on demographic and social structures and beliefs about the health services. In our study, we included maternal ethnicity (majority Kinh or minority ethnic group), occupation (housewife, salaried employee, farmer, or small trader), and education level (at or below primary school: grades 1–5, secondary school: grades 6–9, high school: grades 10–12, or college and higher).

Need factors include indicators of the perceived need for health services based on the health and nutritional status of targeted children. We postulated that having a low birth weight (<2500 grams), undernourished (stunted or wasted) or recently ill (with diarrhea or acute respiratory infection, ARI in the past two weeks, as a proxy for frequent illness) infant/child may urge mothers to seek services. Additional perceived need factors included child age (<6, 6–11.9 or 12–23.0 months) and gender, and number of children under 5 years old in the household.

Enabling factors include resources found within the family and the community, such as socioeconomic status (SES), location of residence, access to health care facilities, and availability of services. In our study, we include exposure to demand generation activities (invitation cards to the MTBT center and A&T promotional billboards and television spots), distance from home to the MTBT center (measured by travel time), and SES. Household SES was estimated using principal components analysis of data on housing conditions and asset holdings, and the first component derived from component scores was used to divide an SES score into tertiles [31, 32].

#### Supply-side factors

Although Andersen identified policy, service structure, and resources as health system characteristics [24], we assumed that health service policy and healthcare system management were similar across all MTBT centers, particularly given the social franchising principles of standardization and uniformity of most organizational elements and our prior knowledge of the Viet Nam health system and the Alive & Thrive intervention. Therefore, we focused on factors related to service structure and resources.

The service structure included a measure of the process of service delivery. Observations of counseling sessions were conducted to assess the extent to which health staff adhered to the standards of care and to evaluate their competence and performance, using an observation checklist based on MTBT service guidelines. At each facility, at least three counseling sessions related to breastfeeding or complementary feeding were observed. A total summative score of technical content delivered and interpersonal communication skills was created and categorized as low, medium, or high counseling skills (based on tertiles).

Resources included human resource factors that may affect service quality. Infrastructure, equipment, and material resources were excluded, assuming uniformity across MTBT centers. Health staff characteristics include age and duration of work at the CHC, IYCF knowledge, job confidence, personal recognition by the government and community, work pressure, salary satisfaction, and supportive supervision. The amount of correct knowledge shared by health staff has been shown to influence the probability of beneficiaries trying the services [33]. Health staff knowledge about IYCF was assessed based on responses to a set of questions, and the overall score of correct knowledge was generated and categorized as low, medium or high. Job confidence, personal recognition, work pressure, salary satisfaction, and supportive supervision are documented to be strongly related to job performance [34] and thereby may influence service utilization. These measures were obtained using a set of questions previously developed for use in rural Haiti [35] and later piloted and adapted for the Viet Nam context. For each question, staff members were asked to indicate their degree of agreement or, where relevant, estimate how often they experienced the circumstance or sentiment in the statement. Each question was scored on a 5-point Likert scale. The total scores for each factor were constructed by summing the item scores and then standardizing the theoretical range of each scale from 0 to 10 for comparability. The scores were categorized as low, medium, or high based on tertiles. For supervision only, scores were divided into two categories: low or high.

### Statistical analysis

Descriptive analyses were used for the patterns of utilization and sample characteristics. Chi-square test was used to assess the difference in the frequency of service use according to child age group. Robust Poisson regression models were applied to analyze the associated factors for different MTBT user groups (one-time user, repeat user, and achiever of minimum number of visits), adjusting for confounding factors such as mother’s age and clustering at the commune level. Independent variables were entered into the models in one step as guided by the conceptual framework. Results are presented as prevalence ratio (PR) with 95% confidence intervals (CI). Population attributable risk (PAR) was estimated in order to determine the additional portion of the population that would achieve the recommended minimum number of visits if fully exposed to that given variable or combination of supply- and demand-side factors. PAR was calculated by the following formula: PAR = Pe (PR-1) / [1 + Pe (PR-1)] where Pe is the prevalence of the exposure (in our case, prevalence of select modifiable variables) and PR is the prevalence ratio of utilization due to select modifiable variables or combination of variables based on the Poisson regression results [36]. All data analyses were performed using Stata version 13 software [37], and p-values <0.05 were considered statistically significant.

## Results

### Characteristics of the study sample

Mean age of mothers was 28 years, and the majority (83%) identified with the Kinh majority ethnicity (Table 1). Half of the mothers had at least nine years of education. The main occupations of mothers were farmers (41%), salaried employees (28%), and small traders (18%). Nearly half of the mothers received invitation cards to MTBT, 28% saw the A&T promotional television spots, and 13% saw an A&T billboard. Nearly three-fourths of the families lived within ten minutes of a MTBT center. While only 4% of children were born low birthweight, 7.7% currently were stunted and 2.5% were wasted, and 31% of children were sick during the last two weeks. Two-thirds of the families had one child under 5 years of age (62%).

**Table 1:** Sample characteristics.

**Demand-side factors (n = 1,008)**			
**Indicators**	**Percent/ Mean ± SD**	**Indicators**	**Percent/ Mean ± SD**
***Maternal predisposing factors*:**		***Need factors*:**	
Age (range: 18 to 45 years)	28.21 ± 5.35	Child age	
Kinh ethnicity	82.84	0–5.9 months	49.80
Education		6–11.9 months	17.46
Primary school	8.04	12–23.9 months	32.74
Middle school	42.56	Child sex	
High school	29.46	Female	47.92
College or higher	19.94	Male	52.08
Main occupation		Low birthweight (<2500 gram)	4.27
Salaried employee	27.58	Child stunting	7.74
Housewife	13.59	Child wasting	2.49
Farmer	41.27	Child illness during last two weeks	30.75
Small trader	17.56	Number of children <5 years	
***Enabling factors*:**		1 child	61.71
Received invitation card to MTBT	44.84	≥ 2 children	38.29
Exposed to promotional TV spot	28.37		
Exposed to promotional bill board	12.70		
Proximity to health facility^1^			
0–5 minutes	36.90		
6–10 minutes	33.43		
11–15 minutes	18.55		
>15 minutes	11.11		
**Supply-side factors (n = 60)**			
**Indicators**	**Percent/ Mean ± SD**	**Indicators**	**Percent/ Mean ± SD**
***CHC staff characteristics*:**		***Service delivery*:**	
Age	41.07 ± 7.06	Counseling skills (range: 86–129)	107.9± 12.1
Gender as female	81.67	IYCF knowledge^2^	5.38± 1.00
Work duration		Confidence^2^	8.11± 0.91
<10 years	38.33	Personal recognition^2^	8.42± 0.98
10–19 years	45.00	Work pressure^2^	6.10± 1.24
≥ 20 years	16.67	Salary satisfaction^2^	7.33± 1.28
		Supportive supervision^2^	8.93± 0.92

^1^Proximixity to health facility is measured by time (in minutes) needed to travel to the facility by motorbike.

^2^Scores for knowledge, confidence, personal recognition, work pressure, salary satisfaction, and supportive supervision are standardized to scales of 0–10 for comparability.

MTBT service providers had a mean age of 41 years; 82% were female, and 62% had at least ten years of work experience (Table 1). Providers were highly confident on their job and experienced positive personal recognition and supportive supervision.

### Patterns of MTBT service utilization

**Fig 3:**
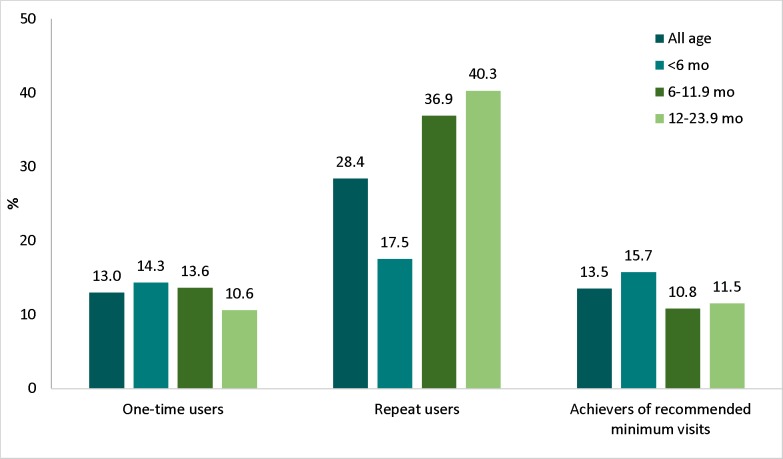
Utilization of IYCF counseling services, by child age group.

Overall, 45.1% of mothers never visited MTBT, 13.0% of mothers visited MTBT only once, 28.4% had repeated visits but did not achieve the minimum number of visits, and 13.5% achieved the recommended minimum number of visits. While repeat users increased significantly with child age, due at least in part to more opportunities for repeated visits, the proportion of children who achieved the minimum number of visits was lower for children 6–23.9 months of age compared to those <6 months (Fig 3).

### Demand-side factors associated with MTBT service utilization

Among the predisposing factors, maternal education (middle school and higher) was strongly associated with higher one-time visits to MTBT (PR: 3.5–3.9) and were more likely to achieve recommended number of visits (PR: 1.8–2.1), compared to mothers with primary school education or lower. Mothers of ethnic minority were close to twice more likely to visit the MTBT at the recommended minimum number of visits (PR: 1.73) compared to Kinh mothers (Table 2). Those who were farmers had 1.5 times higher probability of achieving recommended visits, compared to salaried workers. Maternal age was associated with a small but significant lower repeated use (PR: 0.98).

**Table 2:** Associations between demand- and supply-side factors and categories of IYCF counseling services utilization (groups of MTBT users), compared to never use.

	One-time users (n = 131, 13.0%)		Repeat users (n = 286, 28.37%)		Achievers of recommended minimum visits (n = 136, 13.49%)	
	PR	95% CI	PR	95% CI	PR	95% CI
**Demand-side factors**						
***Maternal predisposing factors***						
Age	0.99	[0.96,1.03]	0.98*	[0.97,0.99]	1.00	[0.98,1.02]
Ethnic minority (ref: Kinh)	1.25	[0.88,1.78]	1.14	[0.99,1.32]	1.73*	[1.09,2.74]
Education (ref: primary school)						
Middle school	3.57*	[1.24,10.23]	1.19	[0.77,1.85]	1.76*	[1.07,2.90]
High school	3.86*	[1.30,11.49]	1.23	[0.72,2.11]	1.88*	[1.10,3.19]
College or higher	3.50*	[1.18,10.33]	1.21	[0.70,2.12]	2.12*	[1.09,4.14]
Main occupation (ref: salaried employee)						
Housewife	1.03	[0.64,1.64]	1.14	[0.87,1.49]	1.05	[0.73,1.52]
Farmer	1.34	[0.94,1.91]	1.14	[0.92,1.42]	1.46*	[1.03,2.07]
Small trader	1.03	[0.67,1.60]	1.04	[0.87,1.25]	1	[0.68,1.49]
***Need factors***						
Child age (months) (ref: 0–5.9)						
6–11.9	1.19	[0.84,1.69]	1.56***	[1.21,2.02]	1.07	[0.61,1.87]
12–23.9	1.01	[0.72,1.42]	1.50**	[1.11,2.04]	1.01	[0.70,1.46]
Child sex as female	1.1	[0.92,1.32]	1	[0.88,1.13]	0.98	[0.77,1.23]
Low birthweight (<2500 gram)	0.5	[0.14,1.81]	0.93	[0.62,1.38]	0.99	[0.61,1.62]
Child stunting	0.86	[0.48,1.54]	0.98	[0.74,1.29]	0.52*	[0.30,0.92]
Child wasting	2.12*	[1.12,4.02]	1.12	[0.72,1.75]	1.62***	[1.22,2.15]
Child Illness	0.74**	[0.61,0.90]	0.91	[0.77,1.07]	0.93	[0.71,1.22]
Number of children <5 ys (ref: 1 child)						
≥ 2 children	1.12	[0.83,1.52]	0.98	[0.81,1.19]	1.25*	[1.00,1.57]
***Enabling factors***						
Received invitation card to MTBT	3.00***	[2.16,4.16]	3.17***	[2.43,4.15]	5.50***	[3.61,8.37]
Exposed to promotional TV spot	1.16	[0.82,1.65]	1.19*	[1.01,1.40]	1.47*	[1.07,2.04]
Exposed to promotional bill board	1.70**	[1.22,2.37]	1.33**	[1.12,1.58]	1.74**	[1.22,2.47]
Household SES (ref: low)						
Middle	1.2	[0.81,1.79]	0.96	[0.81,1.13]	0.95	[0.72,1.25]
High	1.2	[0.79,1.85]	0.99	[0.80,1.23]	0.95	[0.68,1.33]
Proximity to health facility (ref: ≤5 minutes						
6–10 minutes	0.8	[0.55,1.17]	1.1	[0.89,1.35]	1.08	[0.79,1.48]
11–15 minutes	0.65**	[0.47,0.89]	0.88	[0.68,1.14]	0.58**	[0.42,0.82]
>15 minutes	0.69	[0.36,1.32]	0.46**	[0.28,0.76]	0.69	[0.42,1.12]
**Supply-side factors**						
***Service delivery***						
Counseling skills: (ref: low)						
Middle	1.78***	[1.48,2.14]	1.36*	[1.04,1.78]	1.56	[0.98,2.47]
High	1.62***	[1.31,2.00]	1.30*	[1.04,1.63]	1.67**	[1.14,2.44]
***CHC staff characteristics***						
Age (years)	1.01	[1.00,1.01]	1	[0.99,1.01]	1	[0.99,1.02]
Work duration (ref: <10 years)						
10–19 years	0.88	[0.75,1.03]	0.95	[0.84,1.08]	0.73*	[0.57,0.94]
≥ 20 years	0.93	[0.69,1.27]	0.97	[0.75,1.26]	0.68	[0.43,1.09]
Knowledge (ref: low)						
Middle	0.96	[0.79,1.17]	0.9	[0.73,1.11]	0.8	[0.53,1.21]
High	1.02	[0.83,1.26]	1.03	[0.92,1.16]	0.9	[0.70,1.17]
Confident (ref: low)						
Middle	1.09	[0.90,1.32]	1.08	[0.96,1.21]	1.02	[0.84,1.23]
High	1.36**	[1.08,1.71]	1.12	[0.99,1.26]	1.2	[0.88,1.65]
Personal recognition (ref: low)						
Middle	0.94	[0.79,1.11]	0.98	[0.84,1.14]	0.89	[0.68,1.17]
High	0.89	[0.72,1.10]	1.04	[0.92,1.17]	1	[0.84,1.18]
Work pressure (ref: low)						
Middle	0.96	[0.80,1.16]	0.89	[0.79,1.01]	0.71*	[0.52,0.96]
High	0.97	[0.80,1.18]	0.97	[0.83,1.14]	0.76	[0.54,1.07]
Salary satisfaction (ref: low)						
Middle	1.23*	[1.03,1.47]	1.01	[0.89,1.14]	1.25	[0.94,1.67]
High	0.96	[0.84,1.10]	0.88	[0.73,1.06]	1.11	[0.80,1.55]
Supportive supervision (ref: low)						
High	0.92	[0.81,1.05]	1.04	[0.92,1.18]	1.01	[0.79,1.30]

*p<0.05

**p<0.01

***p<0.001.

Among the need factors, mothers with children 6–11.9 months and 12–23.9 months of age were, respectively, 1.5 and 1.6 times more likely to make repeated MTBT visits compared to those with younger children. This finding is probably a reflection of the longer duration of exposure to the program among older children compared to infants less than 6 months of age, which may increase their probability of attending more than once. These age differences were not observed for one-time users or achievers of the recommended minimum number of visits. Compared to mothers with non-wasted children, those who had a wasted child were 1.6 times more likely to have achieved minimum visits. For stunting, the opposite was true: mothers of a stunted child were less likely (PR: 0.5) to have achieved the recommended minimum visits compared to mothers of a non-stunted child. Mothers with a sick child (diarrhea or ARI) were less likely to have ever used the MTBT than mothers of healthy children, potentially due to seeking of treatment or services other than IYCF counselling during illness. Having more than one child under five years of age was also associated with 1.3 times higher of achieving the minimum visits.

Of all the demand (and supply side) factors studied, having received the MTBT invitation card was associated with the highest of MTBT service utilization. Mothers who received invitation cards had 3.0, 3.2 and 5.5 times higher probability of visiting once, repeating visits, and achieving the minimum visits, respectively. Other influential enabling factors included exposure to the promotional TV spots (PR: 1.2 of repeated visits; PR: 1.5 of achieving minimum visits) and to the promotional billboard (PR: 1.7 of one-time visit; PR: 1.3 of repeated visits; PR: 1.7 of achieving minimum visits). Household SES was not associated with use of MTBT, but distance traveled to reach the centers was a major barrier, with mothers traveling more than ten minutes being less likely to achieve the recommended minimum visits (PR: 0.58), and those traveling more than 15 minutes less likely to make repeated visits (PR: 0.47).

### Supply-side factors associated with MTBT service utilization

Service delivery, measured by health staff counselling skills, was an important factor associated with any frequency of MTBT visits (PR: 1.3–1.8 for all 3 groups of users). Given that MTBT service providers are not necessarily new staff, health staff skills may be somewhat known to mothers, even among one-time users; still, better counselling skills resulted in higher utilization, especially for one time users and achievers of recommended minimum visits. Among health staff characteristics, longer duration of work experience and high work pressure were associated with less achieving the minimum number of visits (PR: 0.73 for 10–19 years of work experience; PR: 0.68 for ≥20 years; RR: 0.71–0.76 for high work pressure). High confidence and salary satisfaction were associated with greater probability of one-time visit (PR 1.36 and 1.23, respectively).

### Population attributable risk estimation

**Fig 4:**
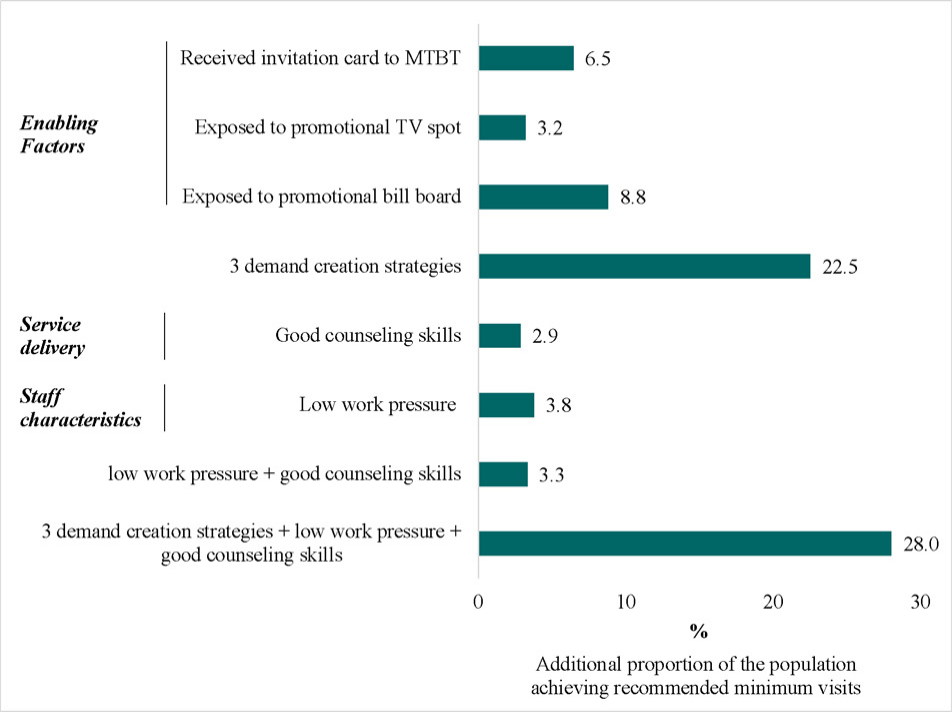
Population attributable risk of achieving the recommended minimum number of visits for select modifiable factors.

Findings from our population attributable risk estimations indicate that if everyone had received an invitation card, an additional 6.1% of the population would have achieved the minimum number of visits compared to the current exposure level (44.8%, Table 1), holding other covariates constant (Fig 4). Similarly, an additional 3.2% and 7.5% of the population would have achieved the minimal number of visits if fully exposed to the promotional TV spot and billboard, respectively. Full exposure to the combination of all three demand generation variables would result in an additional 25.4% achieving the minimum number of visits. Good counseling skills was attributable to an additional 3.2% of the population achieving the minimum number of visits, and low work pressure accounts for 3.6%. Overall, an additional 49% of the population would have achieved the recommended minimum number of visits if the three demand creation approaches had reached all mothers and if good counseling skills and low work pressure had been the norm.

## Discussion

This study brings to light several factors that determine the utilization of IYCF counselling services at MTBT franchises in Viet Nam. Our findings on the demand- and supply-side factors generate insights on which factors, if strengthened, could generate the greatest increases in utilization of the IYCF counseling services offered through a social franchising model in the Viet Nam health system. Our study also contributes to the growing literature documenting the supply- and demand-side factors that influence service provision and use in a variety of contexts and for diverse types of services [38, 39].

Demand generation strategies (i.e., invitation cards to MTBT centers, billboards, and promotional television spots) were key enabling factors that stimulated utilization of IYCF counselling services for first time or repeated users, as well as for those who achieved the recommended number of visits. In particular, dissemination of invitation cards by village health workers was more effective than the other strategies in influencing not only the first visit but continued use of MTBT services. Thus, village health workers should be encouraged to continue this demand generation activity for improved and sustained service utilization. Also, exposure to all three MTBT demand generation activities had an additive effect on service utilization, with an additional 25.4% of the population potentially achieving the minimum number of visits if fully exposed to the three demand generation activities. This suggests that a combination of the demand generation activities could be used to increase MTBT service utilization.

While some studies suggest that lower SES may affect health services utilization due to resource constraints [40–43], this was not a barrier to accessing MTBT services as household SES was not associated with utilization of IYCF counselling services. This is likely because all preventive child health services in Viet Nam, including the MTBT services, are provided free of charge. Consistent with the results from other studies [43–45], we found that physical proximity to services played a role in their utilization. The likelihood of repeated visits to the MTBT centers significantly decreased when the proximity to the facility was greater than 15 minutes, and the likelihood of achieving the minimum number of visits further decreased when the distance to facility was greater than 10 minutes.

Predisposing factors, such as health beliefs, education and other user characteristics, have been found to determine the use of preventive and curative health services [25, 44, 46]. Consistent with findings from other studies on utilization of maternal health service [25, 26, 42, 43] and integrated community case management of childhood illness [28], we found that maternal education was strongly associated with higher MTBT service utilization, particularly one-time use and for achieving minimum number of visits. Mothers with a higher level of education may be more informed and have greater awareness of or capacity to understand health problems experienced by their children as well as the benefits of health care services, which may lead to more effective use of information and to optimal health-seeking behavior [14]. In our study, both ethnic minorities and farmers had higher odds of visiting MTBT. These groups are often marginalized or have less access to services, so it is possible that they were more willing to use MTBT services than those who are of majority ethnicity or engaged in other occupations, when invited or informed about the service. A study found that utilization of antenatal services gradually declined with increasing maternal age, possibly due to greater anxiety, concern, and receptive behavior among younger women [47]; this may have been the case also in our study, where higher maternal age was associated with slightly lower odds of repeated MTBT use. Older women may be less concerned about child feeding, or feel they already have the information they need, therefore less receptive to seeking IYCF counseling on a repeated basis.

Need factors may pose immediate or urgent reasons for seeking health services [48]. Thus, the lack of perceived need would pose a distinct challenge to high service utilization. For IYCF, the perception of need for services is less recognized than it is for illness. In our study, the perceived needs for counselling about child feeding practices were defined by low birthweight (<2500 grams), child stunting or wasting, and recent illness (diarrhea or ARI). Our results showed that mothers with wasted children were more likely to achieve the recommended minimum visits to MTBT, while mothers with stunted or sick children were less likely to achieve the minimum visits or to visit the MTBT even once, respectively. When children are sick, mothers likely seek health services other than nutrition counselling. Because perceived need often involves beliefs about susceptibility, consequences and intervention effectiveness, mothers may seek counselling services for wasting, which is more visible than stunting. It is also possible that wasted children show clear signs of picky eating behavior or anorexia, which could motivate mothers to seek help with child feeding. Although stunting is not always observable by the mother, it is unclear why mothers of stunted children were significantly less likely to continue using MTBT and achieve minimum visits than those whose children were not stunted.

As documented in the literature, we found that better counselling skills (providing appropriate technical information and demonstrating good interpersonal communication skills) was an important factor associated with any frequency of service utilization [8]. In previous studies other indicators of performance of health workers measured by knowledge, skills, incentives, motivation, and aptitude towards their duties were also found to have a strong influence on the use of services [49–51]. Our study, however, did not find an association between work performance indicators such as IYCF knowledge or personal recognition and service use. As expected, heath staff characteristics did not affect one-time use, but longer duration of work and greater work pressure was negatively associated with achieving minimum number of visits. It is possible that staff who have worked for ten or more years become lax in their work, less open to integrating new information and/or practices into their routine, or take on more responsibilities that lead to greater work-related stress, work pressure, or workload, which may compromise the quality of care and influence the quality of interaction with service users. Furthermore, high confidence and salary satisfaction were associated with more one-time use, but not with repeated use nor achieving minimum number of visits, which warrants a closer look at health staff performance within the current salary structure.

Our estimates suggest that an additional 49% of the population could achieve the recommended minimum number of MTBT visits if all three demand creation strategies (invitation cards, promotional spots and bill board) could reach all targeted mothers and if good counseling skills and lower work pressure could become the norm for all health staff. This would represent a 2.5-fold increase in the percentage of targeted women who would complete the minimum number of visits, which would empower them and help them adopt optimal IYCF practices.

Our study has some limitations. Data from cross-sectional household and health staff surveys were used to assess factors associated with counselling service utilization, thus we cannot determine causal relationships between them. For factors related to the need for services, our proxy measures may not reflect the actual needs as perceived by mothers. We did not directly assess the perceived need among mothers in our study, and our measures may not have covered mothers’ perceptions of other needs (e.g., perceptions of poor growth, limited appetite, insufficient breastmilk to fulfill child’s needs, or belief that child may be unhealthy or eating poorly). By applying a comprehensive framework of supply- and demand-side determinants of service utilization, however, we were able to examine various factors at the health system and client levels and identify critical factors influencing IYCF counselling service utilization and the recommended minimum number of visits for the service package.

## Conclusions

Health systems are increasingly paying more attention to nutrition and incorporating preventive nutrition interventions into health services delivery. In this context, especially where health services are often facility-based, optimal use of nutrition interventions and services is critical to ensuring impact. It is, therefore, important to understand and address the factors influencing utilization in order to ensure that beneficiaries receive the full range of nutrition services and maximize their benefits. We have provided a comprehensive investigation of demand- and supply-side factors that influence utilization, focusing on the full range of utilization patterns–from one-time use to repeated use and ultimately achievement of the minimum package of services. Demand-generation strategies and counselling skills were the most important factors associated with any service use; strengthening these two demand -and supply-side factors could markedly improve service utilization with an additional 49% of the population achieving the recommended minimum number of MTBT visits. Given the continued efforts to improve the impact of IYCF interventions and to scale up nutrition through health systems globally, our study contributes robust empirical evidence on potential factors to optimizing facility-based counselling service utilization in Viet Nam. Further research is needed to identify and test methods for increasing and sustaining reach and use of similar services in other contexts.

## Supporting Information

S1 Dataset (DTA)

S2 Dataset (DTA)
